# Bruxism and other jaw loading behaviours are associated with somatic symptoms and psychological distress in orofacial pain-free adults: a cross-sectional study

**DOI:** 10.3389/froh.2025.1622386

**Published:** 2025-08-25

**Authors:** Nontawat Chuinsiri, Peungchaleoy Thammanichanon, Pornputthi Puttaravuttiporn, Wittawat Mongkolchart, Chadatorn Chudet, Sirada Thongraksri

**Affiliations:** ^1^ Institute of Dentistry, Suranaree University of Technology, Nakhon Ratchasima, Thailand; ^2^ Oral Health Centre, Suranaree University of Technology Hospital, Suranaree University of Technology, Nakhon Ratchasima, Thailand; ^3^ Buriram Hospital, Buriram, Thailand; ^4^ Debaratana Hospital, Nakhon Ratchasima, Thailand

**Keywords:** oral parafunction, oral habits, somatoform disorders, medically unexplained symptoms, mental health, temporomandibular joint disorders

## Abstract

**Background:**

Oral behaviours, both functional and non-functional, are commonly reported and can negatively impact oral health. Among orofacial pain patients, non-functional oral behaviours have been observed in association with elevated psychosocial factors. However, the extent to which these findings apply to individuals without orofacial pain remains inconclusive. This study examined the latent constructs of oral behaviours and their associations with somatic symptoms and psychological distress in orofacial pain-free adults.

**Methods:**

This multi-centre cross-sectional study recruited 194 participants who were free of orofacial pain. All participants provided informed consent and completed a set of self-reported questionnaires, including the Oral Behaviour Checklist-21, the Patient Health Questionnaire (PHQ)-15 for assessing somatic symptoms and the PHQ-4 for assessing psychological distress. Exploratory factor analysis (EFA) was conducted to identify latent constructs underlying the observed questionnaire item responses. Correlations between questionnaire scores were assessed using the Spearman's rank correlation test. A *p* value of less than 0.05 was considered statistically significant.

**Results:**

The mean age of participants was 24.6 ± 6.2 years, and 70% were female. Exploratory factor analysis identified three distinct latent constructs of oral behaviours: tooth-contact bruxism, non-tooth-contact bruxism and other jaw loading behaviours. Overall oral behaviours, sleep-state behaviours, awake-state behaviours and the EFA-derived constructs showed statistically significant positive correlations with both somatic symptoms and psychological distress.

**Conclusions:**

In addition to being classified by sleep and awake states, oral behaviours can be grouped into tooth-contact bruxism, non-tooth-contact bruxism and other jaw loading behaviours. The significant correlations observed among oral behaviours, somatic symptoms and psychological distress suggest that individuals reporting frequent oral behaviours should be further evaluated for underlying psychosocial factors, even in the absence of orofacial pain.

## 1 Introduction

Oral behaviours encompass a range of habits that can generally be categorised as either functional or non-functional. Chewing, talking and yawning are examples of functional behaviours, whereas teeth grinding and jaw bracing, also referred to as bruxism, are considered non-functional. These behaviours have the potential to negatively impact oral health (1, 2). Excessive or unbalanced functional behaviours, such as prolonged mouth opening, can lead to the onset of orofacial pain attributed to temporomandibular disorders (TMDs) (3). Similarly, non-functional oral behaviours, such as bruxism, are often associated with painful TMDs as well as with dental attrition (2, 4). Despite their clinical significance, the aetiology of oral behaviours—particularly those classified as non-functional—remains poorly understood (5).

Among the various oral behaviours, bruxism has gained the most interest in research. According to a recent consensus update, bruxism is defined as “a repetitive jaw-muscle activity characterised by clenching or grinding of the teeth and/or by bracing or thrusting of the mandible” (6). Bruxism can occur during either sleep or wakefulness, with global prevalence estimates of 21% for sleep bruxism and 23% for awake bruxism (7). The aetiology of bruxism is multifactorial and proposed to be centrally mediated rather than peripherally driven. Factors involved in its aetiology include stress, anxiety, genetic susceptibility, neurochemical imbalances, reduced airway patency during sleep, as well as external factors such as smoking and substance use (8, 9).

Multiple studies have reported positive associations between oral parafunctional behaviours and heightened psychological distress in patients experiencing painful TMDs (5, 10–13). It is not uncommon for individuals to express their underlying psychological distress through physical or somatic symptoms, a phenomenon known as somatisation (14, 15). Common manifestations of somatisation include headaches, dizziness and sleep disturbances, which can be assessed using validated tools such as the Patient Health Questionnaire (PHQ)-15 (16). Accordingly, it could be hypothesised that oral behaviours, such as bruxism, may represent somatic symptoms associated with psychological distress. However, given the chronic nature of painful TMDs, a bidirectional relationship must be considered, whereby oral behaviours may serve as maladaptive coping mechanisms in response to persistent orofacial pain (5). The current body of research is limited by a paucity of studies involving orofacial pain-free individuals; this limitation creates a gap in understanding the precise relationship between oral behaviours, somatisation and underlying psychological distress, independent of pain as a confounding factor. In addition, functional oral behaviours have been relatively underexplored, compared to their non-functional counterparts.

The present study, therefore, aimed to examine the association between oral behaviours, somatic symptoms and psychological distress in an orofacial pain-free adult population using validated instruments. Distinct subscales of oral behaviours, including sleep-state, awake-state, functional and non-functional, were also considered in the analyses.

## 2 Materials and methods

This study complied with the Strengthening the Reporting of Observational Studies in Epidemiology Guidelines (17).

### 2.1 Setting and participants

In this cross-sectional study, orofacial pain-free participants aged 18–44 years were recruited between March and November 2024 from the Oral Health Centre at Suranaree University of Technology Hospital (Nakhon Ratchasima, Thailand), two private dental clinics in Nakhon Ratchasima, the Dental Department at Buriram Hospital (Buriram, Thailand) and a private dental clinic in Buriram. Participants were excluded if they had a craniofacial syndrome, a genetic disorder affecting pain perception or were undergoing active orthodontic treatment. Informed consent was obtained from all eligible participants prior to data collection. The study was approved by the Human Research Ethics Committee of Suranaree University of Technology (Approval no. EC-67-13) and the Human Research Ethics Committee of Buriram Hospital (Approval no. BR0033.102.1/17).

### 2.2 Data collection

After obtaining informed consent, trained research staff provided participants with questionnaires to complete independently without further instructions to reduce variability between study sites. Demographic data, including sex and age, were also collected. The questionnaires for assessing psychological distress, somatic symptoms and oral behaviours were based on Axis II of the Diagnostic Criteria for TMDs (DC/TMD) (18). The Thai versions of these questionnaires, translated in accordance with the International Network for Orofacial Pain and Related Disorders Methodology (INfORM) standards and publicly available on the INfORM website (https://inform-iadr.com/), were utilised in this study.

The PHQ-4, a 4-item questionnaire, was used to assess psychological distress. Each item is rated on a 4-point Likert scale, ranging from 0 (not at all) to 3 (nearly every day). Total scores span from 0–12, with scores of 3, 6 and 9 representing cut-off points for mild, moderate and severe levels of psychological distress, respectively (19).

Somatic symptoms were assessed using the PHQ-15, which consists of 15 items addressing common somatic symptoms. Each item is rated on a 3-point Likert scale, ranging from 0 (not bothered) to 2 (bothered a lot). Total scores span from 0–30, with scores of 5, 10 and 15 representing cut-off points for low, medium and high levels of somatic symptom severity, respectively (16).

Oral behaviours were assessed using the 21-item Oral Behaviour Checklist (OBC-21). The OBC-21 comprises two items assessing sleep-state behaviours and 19 items assessing awake-state behaviours, both functional and non-functional. Each item measures the frequency of a specific oral behaviour on a 5-point Likert scale, ranging from 0 (none of the time) to 4 (four to seven nights per week or all of the time). Conventionally, frequency scores can be calculated as follows: OBC-21 (sum of all items), OBC-sleep (sum of the two sleep-state items) and OBC-awake (sum of the 19 awake-state items) (20, 21).

### 2.3 Data analyses

Data analyses were performed using GraphPad Prism version 10.4 (GraphPad Software, Boston, MA, USA), unless otherwise specified. Descriptive statistics for numerical variables were calculated and presented as the mean, standard deviation (SD), first quartile (Q1), median and third quartile (Q3). Categorical variables were summarised as frequency counts and percentages. The Shapiro–Wilk test was employed to assess the normality of data. Comparisons of age, which was non-normally distributed, and ordinal variables, including all questionnaire scores, between males and females were conducted using the Mann–Whitney *U* test. Categorical variables were compared between sexes using the Fisher's exact test.

Exploratory factor analysis (EFA) was employed to identify latent constructs (factors) underlying the observed variables and to group related variables accordingly (22). The suitability of the dataset for EFA was first evaluated using the Kaiser-Meyer-Olkin (KMO) measure of sampling adequacy and the Bartlett's test of sphericity. The factor_analyzer package (https://factor-analyzer.readthedocs.io/en/latest/index.html) in Python version 3.11 was used to conduct EFA on 40 questionnaire items from the PHQ-4, PHQ-15 and OBC-21. The number of factors to retain was determined based on the Kaiser's rule, whereby factors with eigenvalues greater than one were retained. Factors were extracted using a minimum residual method, followed by oblique rotation (Promax) to facilitate interpretation. Items with factor loadings exceeding 0.40 were considered meaningful contributors to a factor. The internal consistency of each identified factor was evaluated using Cronbach's alpha, with a value of 0.70 or higher indicating acceptable reliability.

Raw and partial correlations between ordinal variables were assessed using the Spearman's rank correlation test, implemented via the pingouin package (https://pingouin-stats.org/build/html/index.html) in Python. Assuming a two-tailed significance level of 0.05, a power of 80% and an expected Spearman's rho coefficient of 0.2, a minimum of 194 participants was required to detect a small effect size (23). A *p* value of less than 0.05 was considered statistically significant for all tests.

## 3 Results

### 3.1 Characteristics of study participants

Participant characteristics are summarised in Table 1 for the total sample (*n* = 194) and stratified by sex. No significant sex differences were observed for any of the variables. In addition, the Spearman's rank correlation test revealed no significant correlations between age and any of the questionnaire scores.

**Table 1:** Summary of participant characteristics and between-sex differences.

Variable		Total (*n* = 194)	Male (*n* = 58)	Female (*n* = 136)
Age	Mean ± SD	24.6 ± 6.2	24.9 ± 6.2	24.5 ± 6.2
	Q1	19.8	20.0	19.0
	Median	23.0	23.0	22.0
	Q3	29.3	29.3	29.3
PHQ-4	Mean ± SD	1.7 ± 1.9	1.5 ± 1.9	1.7 ± 1.9
	Q1	0	0.0	0.0
	Median	1.0	1.0	1.0
	Q3	2.0	2.0	3.0
	None-to-minimal (*n*, % of column)	149 (76.8)	48 (82.8)	101 (74.3)
	Mild (*n*, % of column)	35 (18.0)	8 (13.8)	27 (19.9)
	Moderate (*n*, % of column)	8 (4.1)	1 (1.7)	7 (5.1)
	Severe (*n*, % of column)	2 (1.0)	1 (1.7)	1 (0.7)
PHQ-15	Mean ± SD	5.0 ± 5.1	4.2 ± 5.0	5.4 ± 5.1
	Q1	1.0	1.0	1.0
	Median	4.0	3.0	4.0
	Q3	7.3	5.5	8.0
	None-to-minimal (*n*, % of column)	115 (59.3)	40 (69)	75 (55.1)
	Low (*n*, % of column)	41 (21.1)	10 (17.2)	31 (22.8)
	Medium (*n*, % of column)	28 (14.4)	5 (8.6)	23 (16.9)
	High (*n*, % of column)	10 (5.2)	3 (5.2)	7 (5.1)
OBC-21	Mean ± SD	22.1 ± 10.0	23.2 ± 10.4	21.7 ± 9.8
	Q1	15.0	16.8	13.3
	Median	21.0	23.0	21.0
	Q3	29.0	30.0	29.0
OBC-sleep	Mean ± SD	3.0 ± 2.3	3.2 ± 2.4	3.0 ± 2.2
	Q1	1.0	1.0	1.0
	Median	3.0	3.0	3.0
	Q3	4.0	5.0	4.0
OBC-awake	Mean ± SD	19.1 ± 8.6	20.0 ± 9.1	18.7 ± 8.4
	Q1	13.0	13.8	12.3
	Median	19.0	19.5	19.0
	Q3	25.0	25.3	25.0

PHQ, patient health questionnaire; OBC, oral behaviour checklist; SD, standard deviation; Q1, first quartile; Q3, third quartile.

### 3.2 Distinct latent constructs of oral behaviours were identified through EFA

**Figure 1:**
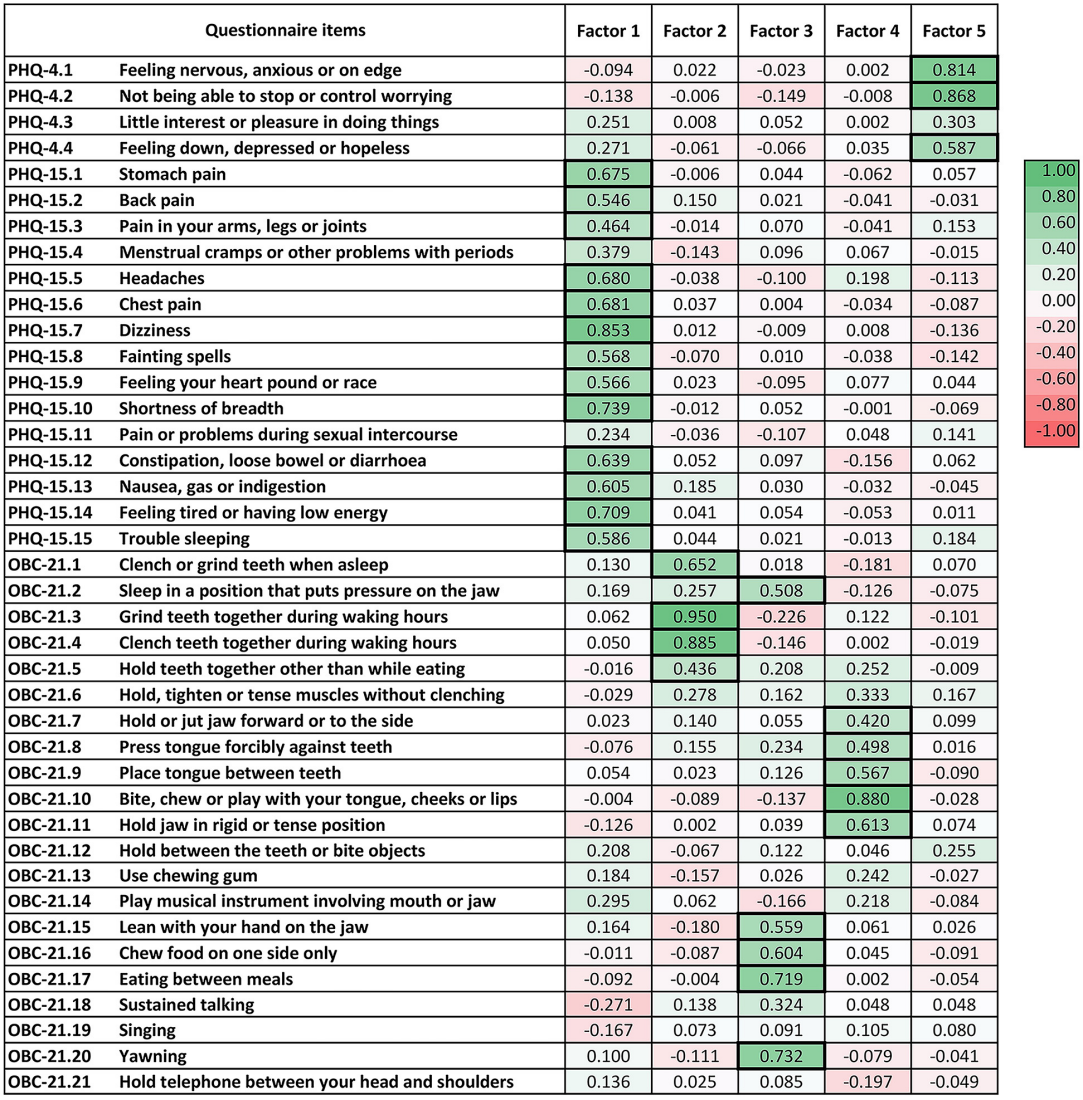
Exploratory factor analysis of items from the patient health questionnaire (PHQ)-4, PHQ-15 and oral behaviour checklist (OBC)-21. Factors 1 to 5 demonstrate acceptable reliability with Cronbach's alpha >0.7. Factor loadings above 0.4 are highlighted with a bold frame.

The KMO value was 0.84, exceeding the recommended value of 0.6, and Bartlett's test was significant (chi-squared = 3,210.25, *p* < 0.05), indicating that EFA was appropriate for the dataset. Nine factors with eigenvalues greater than one were identified in the EFA, accounting for 49% of the total variance. Figure 1 presents a heatmap of the factor loadings for each questionnaire item.

Factor 1 consisted most of the items from the PHQ-15, with the exception of item 4 (menstrual cramps or other problems with your periods) and item 11 (pain or problems during sexual intercourse), which showed loadings greater than 0.4 on Factors 9 and 8, respectively. The Cronbach's alpha for both Factor 1 and the entire PHQ-15 was 0.9.

Factors 2, 3 and 4 constituted item groupings from the OBC-21; each factor represented a distinct latent construct within the domain of oral behaviours. The Cronbach's alpha for the entire OBC-21 was 0.86. Factor 2 included four OBC-21 items related to tooth-contact bruxism (item 1: clench or grind teeth when as sleep, item 3: grind teeth together during waking hours, item 4: clench teeth together during waking hours and item 5: press, touch or hold teeth together other than while eating), with a Cronbach's alpha of 0.79. Factor 3 encompassed five OBC-21 items related to jaw loading behaviours other than bruxism (item 2: sleep in a position that put pressure on the jaw, item 15: lean with your hand on the jaw, item 16: chew food on one side only, item 17: eating between meals and item 20: yawning), with a Cronbach's alpha of 0.73. Factor 4 comprised five OBC-21 items related to bruxism without tooth contact (item 7: hold or jut jaw forward or to the side, item 8: press tongue forcibly against teeth, item 9: place tongue between teeth, item 10: bite, chew or play with your tongue, cheeks or lips and item 11: hold jaw in rigid or tense position), with a Cronbach's alpha of 0.75.

Factor 5 included items 1, 2 and 4 from the PHQ-4. The Cronbach's alpha for both Factor 5 and the entire PHQ-4 was 0.74.

Factors 6 and 8 had Cronbach's alpha values below 0.7. Factors 7 and 9 each included only one item. No factors emerged with items from different questionnaires.

### 3.3 Bruxism and other jaw loading behaviours were associated with somatic symptoms and psychological distress

**Figure 2:**
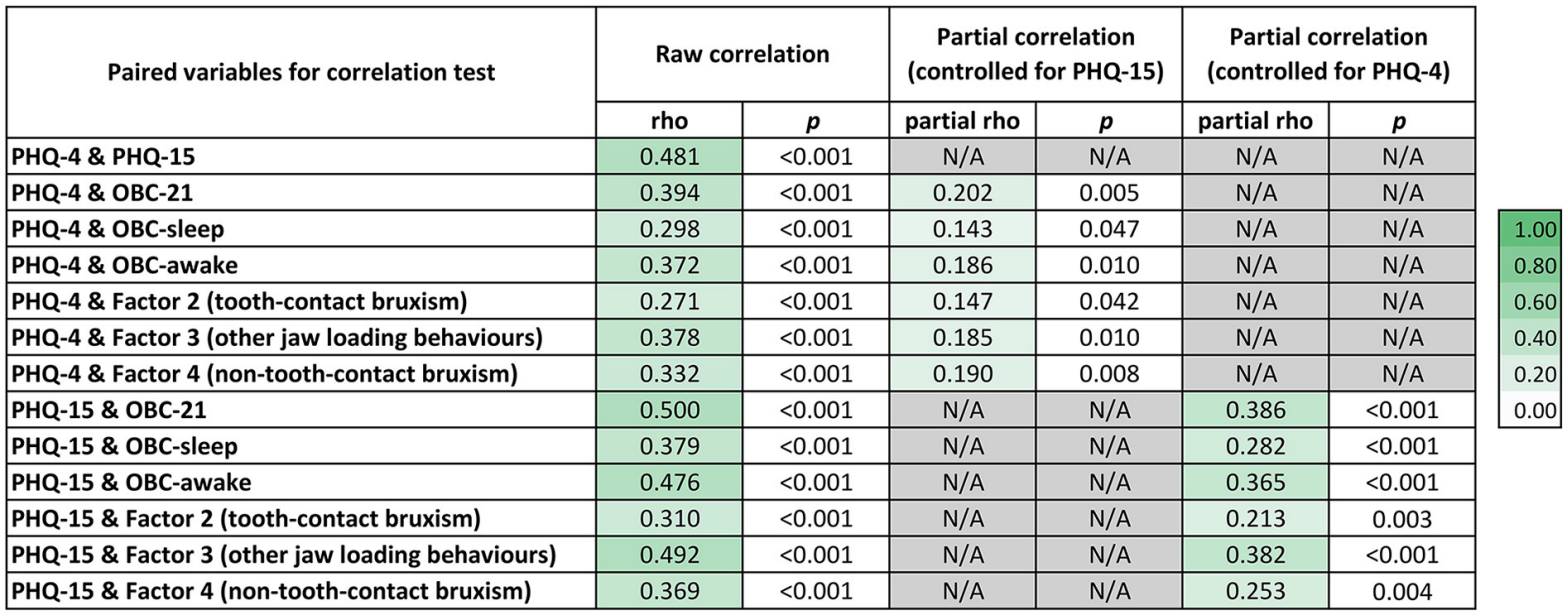
Correlation among oral behaviours (OBC-21), psychological distress (PHQ-4) and somatic symptoms (PHQ-15). Raw Spearmans rho coefficients and their corresponding *p* values are presented for each pairwise correlation. Partial correlation coefficients, adjusted for PHQ-15 and PHQ-4, are also presented where relevant. Colour intensity reflects the strength of the correlation, with darker shades representing stronger correlations.

Results from the Spearman's rank correlation test are shown in Figure 2. A weak but statistically significant positive correlation was found between PHQ-4 scores and OBC-21 scores—including all subscales and latent constructs previously identified through EFA—when controlling for PHQ-15. When controlling for PHQ-4, a moderate significant positive correlation was observed between PHQ-15 scores and the overall OBC-21 score, as well as with the OBC-awake subscale and Factor 3. In addition, PHQ-15 scores were weakly and positively correlated with the OBC-sleep subscale, Factor 2 and Factor 4.

## 4 Discussion

This study investigated the associations between oral behaviours, somatic symptoms and psychological distress in a sample of adults without orofacial pain. Through EFA, three distinct latent oral behaviour constructs were identified: (i) tooth-contact bruxism, (ii) non-tooth-contact bruxism and (iii) other jaw loading behaviours. Overall oral behaviours, as well as sleep-state behaviours, awake-state behaviours and the EFA-derived constructs, showed weak to moderate positive correlations with both somatic symptoms and psychological distress.

### 4.1 Oral behaviour latent constructs

Previous studies in patients with TMDs have identified two latent constructs, namely non-functional and functional oral behaviours, within the 19 awake-state items of the OBC-21 (1, 24). A study in an Italian sample found that non-functional oral behaviours consisted of items 3–11, excluding item 10, with factor loadings above 0.4 (1). By comparison, a study in a Chinese sample included items 3–10, excluding item 4, suggesting slight cross-cultural differences (24). The current bruxism definition aligns with the non-functional items 1, and 3–11 of the OBC-21. In the present study, we adopted a slightly different approach, as sleep and awake bruxism can simultaneously occur and share common risk factors (10, 25–27), and may therefore be underpinned by the same construct. We incorporated both sleep-state and awake-state OBC-21 items in the EFA and likewise identified non-functional oral behaviour constructs. However, our analyses partitioned these non-functional oral behaviour items into two distinct constructs, which we termed the “tooth-contact bruxism” construct (items 1, 3, 4 and 5) and the “non-tooth-contact bruxism” construct (items 7–11). The clinical relevance of these novel constructs needs to be further explored.

In addition to the two bruxism constructs, we identified a third construct, including behaviours that impose excessive or unbalanced loading on the jaw, specifically items 2, 15–17 and 20. These items have been categorised as functional behaviours in previous studies; however, between-study variation has been observed regarding which items are considered to constitute these functional behaviours, with only item 17 (eating between meals) being consistently included. Of particular interest is our identification of item 16 (chewing food on one side only) as part of this construct, whereas this item was absent in previous studies (1, 24). This finding suggests potential cross-cultural differences, with unilateral chewing preference possibly being more prevalent among Thais. Another possible explanation is that our sample comprised orofacial pain-free individuals, whereas previous studies included TMD patients, who have been shown to exhibit lower functional behaviours (1). It is plausible that, in TMD patients, chewing becomes more balanced to mitigate pain (28), leading to less frequent reporting of item 16. The functional nature of this third construct, particularly yawning (29, 30), remains equivocal. Therefore, based on the balance of evidence, we termed this construct “other jaw loading behaviours”.

### 4.2 Associations among oral behaviours, somatic symptoms and psychological distress

In the present study, EFA did not identify any constructs comprising items from different questionnaires, suggesting that each questionnaire measures a unique psychosocial aspect. This finding refutes our initial hypothesis that some oral behaviours may represent somatic symptoms associated with psychological distress. Nevertheless, significant correlations between these questionnaires scores indicated that these distinct constructs were nonetheless related. The effect sizes—correlation coefficients—reported in our studies ranged from weak to moderate, which is consistent with previous research investigating the correlation between oral behaviours and psychological factors (10, 12, 13). However, the interpretation of effect sizes has been suggested to be field-specific (31). Given that specific guidelines for correlation effect sizes related to the temporomandibular joint and masticatory muscles have yet to be established, all data should be interpreted with caution.

As previously reported in patients with TMD pain (11, 12) and in healthy adolescents (32), we also found that a range of oral behaviours was associated with somatic symptoms and psychological distress in orofacial pain-free adults. Given that this association is evident even in healthy individuals, it is possible that psychological distress alone may contribute to the self-reporting of oral behaviours, independent of any pain-related coping mechanism. However, in individuals with TMD pain, the frequency of non-functional oral behaviours may be further elevated (1, 11). Although the precise mechanisms underlying this relationship remain unclear, several explanations have been proposed. One possibility is that psychological distress leads to increased tension and activity in the masticatory muscles, manifesting as bruxism (33–36). This is supported by previous studies showing that experimental stress increases electromyographic activity of the masseter muscles in both TMD patients and healthy participants (37, 38). Another previously proposed explanation is that individuals with higher levels of psychological distress and somatic symptoms may exhibit heightened interoceptive awareness, making them more likely to notice and report oral behaviours, even if the actual behaviours do not significantly deviate from the norm (13). Neurobiological mechanisms may also play a role. Central sensitisation—a condition in which the central nervous system becomes hypersensitive to both painful and non-painful stimuli—is frequently associated with emotional distress, including anxiety and depression, as well as physical symptoms such as poor sleep and non-functional oral behaviours like teeth grinding and clenching (39, 40). This mechanism may explain the observed correlations among psychological distress, somatic symptoms and oral behaviours in the present study.

Interestingly, somatic symptoms showed a moderate correlation with other jaw loading behaviours, stronger than that observed for the bruxism constructs. This could be due to the fact that individuals with high levels of somatic symptoms tend to exhibit heightened bodily awareness and conscious monitoring (41, 42). Jaw loading behaviours—such as yawning or certain sleeping positions—are more likely to be noticed and reported by individuals, in contrast to sleep-related or non-functional oral behaviours, which typically occur outside of conscious awareness.

### 4.3 Implications for research and practice

Although the questionnaire scores were significantly correlated, EFA demonstrated that the PHQ-4, PHQ-15 and OBC-21 represented distinct constructs and should be retained as separate instruments in clinical and research settings. For the OBC-21 in particular, latent constructs were identified, suggesting that subscale-level interpretation should be considered in practice. Some items within the OBC-21, such as item 14 (play a musical instrument involving the use of the mouth or jaw), lacked a meaningful underlying construct, which is consistent with previous research (1, 24). Such items may be candidates for removal in the development of an abbreviated version of the questionnaire. Another important implication is that patients who report significant detrimental oral behaviours should be assessed for psychosocial characteristics, including psychological distress and somatic symptoms, even in the absence of orofacial pain. Given the significant associations observed, early identification of these psychosocial risk factors may contribute to the prevention of future orofacial disorders.

### 4.4 Strengths and limitations

To our knowledge, this is the first multi-centre study to examine oral behaviour constructs and their relationship with somatic symptoms and psychological distress in adults without orofacial pain. Excluding individuals with orofacial pain allowed for a clearer evaluation by eliminating pain as a potential confounding factor. In addition, participants undergoing active orthodontic treatment, which was previously reported to influence bruxism (43, 44), were also excluded.

Some limitations can be appreciated in this study. The cross-sectional design limits the ability to establish causality between oral behaviours, somatic symptoms and psychological distress. Multiple correlation tests were performed on the dataset, which may increase the risk of Type I errors; therefore, findings should be interpreted cautiously, considering both effect sizes and *p* values. Additionally, all data were self-reported, which may introduce biases such as social desirability or recall bias. Objective measures of oral behaviours were not employed, which may limit the accuracy of behavioural assessment. Lastly, the characteristics of our sample—70% female participants with a mean age of 24.6 ± 6.2 years—may limit the generalisability of the findings.

### 4.5 Conclusions

In addition to the conventional distinction between sleep and awake states, self-reported oral behaviours can be grouped into tooth-contact bruxism, non-tooth-contact bruxism and other jaw loading behaviours. The frequency of self-reported oral behaviours has been shown to correlate with levels of somatic symptoms and psychological distress. Therefore, patients reporting significant oral behaviours should be further assessed for underlying psychosocial factors, regardless of the presence of orofacial pain.

## Data Availability

The raw data supporting the conclusions of this article will be made available by the authors, without undue reservation.
